# The potential of arts therapies in Parkinson's disease rehabilitation: A comprehensive review

**DOI:** 10.1016/j.heliyon.2024.e35765

**Published:** 2024-08-03

**Authors:** Yiyuan Li, Xuexing Luo, Aijia Zhang, Fangtian Ying, Jue Wang, Guanghui Huang

**Affiliations:** a Faculty of Humanities and Arts, Macau University of Science and Technology, Avenida Wai Long, Taipa, Macau, 999078, China; b State Key Laboratory of Quality Research in Chinese Medicines, Macau University of Science and Technology, Avenida Wai Long, Taipa, Macau, 999078, China; c Faculty of Chinese Medicine, Macau University of Science and Technology, Avenida Wai Long, Taipa, Macau, 999078, China; d Guangdong-Hong Kong-Macao Joint Laboratory for Contaminants Exposure and Health, Guangzhou, Guangdong, China; e Zhejiang University, Hangzhou, 310027, China; f Zhuhai M.U.S.T. Science and Technology Research Institute, Zhuhai, Guangdong, China

**Keywords:** Parkinsonian disorders, Rehabilitation, Arts therapies, Complementary therapies, Psychotherapy

## Abstract

**Background and purpose:**

Parkinson's disease (PD) causes a decline in motor function, cognitive decline, and impacts the mental health of patients. Due to the high cost and side effects of conventional treatments, the medical community has begun to explore safer and more cost-effective alternative therapies. In this context, arts therapies have gained increasing attention as innovative treatments. This review plans to explore the role and potential of various arts therapies in the rehabilitation of PD patients by analyzing existing literature and case studies.

**Methods:**

This review comprehensively searched the literature in several databases, including PubMed, Embase, Cochrane Library, Web of Science, and China National Knowledge Infrastructure, to assess the effectiveness of different arts therapies in the rehabilitation of patients with PD.

**Results:**

From 3440 articles screened, 16 met the inclusion criteria. These studies included a variety of therapies, including music, meditation, yoga, art, dance, theatre, video games and play therapy. These different types of arts therapies had a positive impact on the motor, psychological and cognitive rehabilitation of PD patients, respectively.

**Conclusion:**

The existing literature highlights the great potential of arts therapies in the rehabilitation of people with PD, further confirming the efficacy of arts therapies in enhancing the motor, psychological and cognitive rehabilitation process of people with PD. In addition, this review identifies research gaps in the use of color therapy in PD rehabilitation and highlights the need for further exploration of various arts therapies modalities.

## 1 Introduction

Parkinson's disease (PD) is the second most prevalent neurodegenerative disorder, trailing only Alzheimer's disease in its frequency [1]. A higher susceptibility has been observed in males compared to females [2]. Globally, approximately 6 million individuals are diagnosed with PD, a number projected to double by 2030 [3,4]. While predominantly manifesting in the elderly, PD can also affect younger individuals. The primary physical manifestations of PD include postural instability, resting tremor, and bradykinesia [5,6]. In addition to the decline in motor functions, patients may also experience cognitive and emotional disorders impacting speech and communication abilities, restricting daily activities and social interactions [7,8]. Psychological responses such as loss of independence, depression, sleep disturbances, psychosis, and anxiety [9] can significantly impair patients' quality of life and well-being [10,11].

Due to the incurable nature of PD, patients rely on ongoing support and therapy for symptom management [12]. Traditional treatments, including deep brain stimulation (DBS) surgery and medication, are commonly used to manage symptoms in patients with PD. DBS surgery modifies electrical activity in the brain to address movement issues, while medication aims to increase dopamine levels to alleviate symptoms. However, these treatments have drawbacks such as invasiveness, risk, and cost associated with DBS surgery, and the diminishing effectiveness and adverse effects of medications over time [13]. Consequently, there is an increasing need to investigate safer and more economically effective therapeutic alternatives.

Arts therapies offers a comprehensive and compassionate treatment option for those with PD [14,15]. Arts therapies is a novel non-medical method that improves physical coordination, cognitive capacities, and personal emotions through creative activities like music, dancing, and painting. Arts therapies described by the British Association of Art Therapists as "a type of psychotherapy in which the artistic medium is the primary means of communication" [16]. Arts therapies not only improve physical functioning but also provide essential psychological support for patients coping with the challenges of a chronic illness [17,18]. Furthermore, arts therapies facilitate increased social connection and community participation through group activities that foster interaction, cooperation, and a sense of belonging, thereby reducing isolation and enhancing social skills and self-esteem [19]. With its emphasis on non-pharmacological intervention, arts therapies have garnered attention for their potential in the rehabilitation of PD patients, offering a comprehensive and humanized approach to intervention [20,21].

Therefore, the purpose of this review is to investigate the potential of arts therapies in PD rehabilitation by analyzing the existing literature and case studies to examine and analyze the use of arts therapies in PD interventions and its benefits in improving PD symptoms. In addition, we explore the variability and differences that exist between these arts therapies in the treatment of PD in an attempt to identify existing research gaps in the field of arts therapies for PD rehabilitation and suggest future research directions.

## 2 Materials and methods

### 2.1 Data sources and search strategies

The objective of this scholarly endeavor was to rigorously explore the efficacy and role of arts therapies modalities in the rehabilitation of patients afflicted with PD. To achieve this, a comprehensive and systematic literature search was conducted across multiple esteemed databases, including PubMed, Embase, Cochrane Library, Web of Science, and the China National Knowledge Infrastructure (CNKI). The search aimed at identifying relevant articles indexed under medical subject headings (MeSH) such as “Art Therapy”, “Music Therapy”, “Dance Therapy”, “Psychodrama”, “Play Therapy”, “Color Therapy”, “Video games”, “Yoga”, “Meditation”, “Parkinsonian Syndrome”, “Parkinsonian Syndromes”, “Parkinsonian Diseases”, “Parkinsonism”, “Parkinson's Disease”, “Paralysis Agitans” and “Parkinsonian Disorders”. The inclusion criteria, as outlined in Table 1, were meticulously formulated to incorporate studies that specifically address the application of arts therapies in PD, aligning with the aforementioned MeSH terms. Furthermore, the temporal scope of this literature review covered the period from January 2000 to December 2023, and non-English language literature was excluded. Setting time and language limits is intended to allow the research team to focus on the latest relevant research within the limits of their language skills, to ensure the quality and validity of the research [22].

**Table 1:** Search strategies for English databases or Chinese databases.

Number	Search Terms
#1	Art therapy [MeSH*]
#2	Music Therapy [MeSH*]
#3	Dance Therapy [MeSH*]
#4	Play therapy [MeSH*]
#5	Psychodrama [MeSH*]
#6	Color Therapy [MeSH*]
#7	Video Games [MeSH*]
#8	Yoga [MeSH*]
#9	Meditation [MeSH*]
#10	#1 OR #2 OR #3 OR #4 OR #5 OR #6 OR #7 OR #8 OR #9
#11	Parkinsonian Disorders [MeSH*]
#12	Parkinsonian Syndrome [MeSH*]
#13	Parkinsonian Syndromes [MeSH*]
#14	Parkinsonian Diseases [MeSH*]
#15	Parkinsonism [MeSH*]
#16	Paralysis Agitans [MeSH*]
#17	Parkinson's Disease [MeSH*]
#18	#11OR #12 OR #13 OR #14 OR #15 OR #16 OR #17
#19	#10 AND #18
#20	Yishu Zhiliao (Art therapy)
#21	Yinyue Liaofa (Music Therapy)
#22	Wudao Liaofa (Dance Therapy)
#23	Youxi Liaofa (Play Therapy)
#24	Xiju Liaofa (Psychodrama)
#25	Huihua Liaofa (Color Therapy)
#26	Youxi Liaofa (Video Games)
#27	Yujia Liaofa (Yoga)
#28	Mingxiang Liaofa (Meditation)
#29	#20 OR #21 OR #22 OR #23 OR #24 OR #25 OR #26 OR #27 OR #28
#30	Pajinsen Zonghezheng (Parkinsonian Syndromes)
#31	Pajinsen Bing (Parkinsonian Disorders)
#32	Pajinsen (Parkinsonism)
#33	Pajinsenshi Bing (Parkinson's Disease)
#34	Zhenchanmabi (Parkinson's Disease)
#35	#30 OR #31 OR #32 OR #33 OR #34
#36	#29 AND #35

Abbreviation: MeSH = Medical Subject Headings.

The search method was derived from the PubMed database and applied to other databases. The formula is used consistently across all databases: Art therapy [MeSH*] OR Music therapy [MeSH*] OR Dance therapy [MeSH*] OR Play therapy [MeSH*] OR Psychodrama [MeSH*] OR Color therapy [ MeSH*] OR Video Games [MeSH*] OR Yoga [MeSH*] OR Meditation [MeSH*] AND Parkinsonian Disorders [MeSH*] OR Parkinsonian Syndrome [MeSH*] OR Parkinsonian Syndromes [MeSH*] OR Parkinsonian Diseases [MeSH*] OR Parkinsonism [MeSH*] OR Paralysis Agitans [MeSH*] OR Parkinson's Disease [MeSH*].

### 2.2 Inclusion and exclusion criteria

The inclusion criteria for the study can be specifically categorized into three points: 1. Patients who have been diagnosed with PD and are using arts therapies as their primary intervention. Arts therapies may include a range of modalities, including, but not limited to, music, painting, dance, theatre, and drama therapy. 2. The article provides relevant data to support and validate the use of arts therapies in patients with PD. 3. Peer-reviewed literature, which may include, but is not limited to, randomized controlled trials (RCTs), cohort studies, reviews, meta-analyses, and cross-sectional analyses of studies.

On the basis of the inclusion criteria, we determined the exclusion criteria for this review: 1. Non-peer-reviewed literature, studies that did not use arts therapies-based interventions, and studies of individuals without a diagnosis of PD were excluded. This selectivity ensured the inclusion of studies with robust methodologies and relevant outcomes, thus providing reliable evidence of the effectiveness of arts therapies in the treatment of PD symptoms. 2. Exclude literature in the ‘grey literature’ category, such as unpublished manuscripts or conference abstracts. 3. Exclude literature not written in English. The exclusion of non-English articles and grey literature was considered a limitation, but it was necessary for the review team to reduce the complexity and heterogeneity that can occur during the literature screening and data extraction process and to ensure that the review team was able to conduct a more efficient and in-depth analysis within the limits of their linguistic abilities [22].

### 2.3 Data extraction and quality evaluation methods

In this study, we implemented a comprehensive literature screening and evaluation process, executed by two independent reviewers, LYY and LXX, to ensure objectivity and accuracy in our research. Initially, LYY was responsible for downloading and conducting a preliminary review of the screened literature, aiming to exclude any documents irrelevant to our research theme. Subsequently, the preliminarily screened relevant documents were handed over to LXX for a more thorough eligibility evaluation, and the LYY and LXX identified the final analyses for inclusion in the literature. Only documents mutually agreed upon by LYY and LXX were included in the final analysis.

During the data extraction process, LYY and LXX created four different forms, each with a specific function, to systematically organize and analyze the data collected. The main purpose of developing these tables was to organize and analyze the data in a systematic and standardized way, thus increasing the transparency, systematization and scientifically rigorous nature of this review. The first table was designed to present essential data extracted from each piece of literature, including types of art, types of study, study objectives, participants, methodology, results, and analysis. This table aims to facilitate an immediate understanding of the key components of each study, providing a quick and comprehensive overview of the collected data. The second table is dedicated to evaluating the quality of the literature. It utilizes a checklist derived from the JBI guidelines to assess the methodological quality of studies [23]. This table aims to ensure that the included studies maintain high scientific rigor while avoiding bias associated with low-quality studies. The third table is dedicated to extracting detailed data on patient samples, intervention and control group methods, and outcomes, particularly from RCTs. This table uses the PICO(S) model to provide a structured and comprehensive analysis of randomized controlled trials, which aims to improve the clarity and organization of the study data and to lay a solid foundation for subsequent analysis and discussion in this review [24]. The fourth table is dedicated to illustrating subgroups within different arts therapies. Its purpose is to reveal commonalities and differences among treatment outcomes in order to provide transparency and scientific support for subsequent analysis in this review. The fifth table specifically targets the extraction of rehabilitation effects resulting from various arts therapies for PD patients. Its aim is to highlight how various arts therapies can improve rehabilitation outcomes for PD patients.

To ensure the quality of the selected literature, this review utilized the JBI critical appraisal tools to conduct an evaluation related to the quality of the literature [23]. The quality assessment of the final included literature was conducted using the scoring system provided by the JBI guidelines. This system allocates one point for each criterion fully met, ‘Yes’, and zero points for criteria ‘no’ and ‘unclear’. This scoring method aids in the horizontal comparison of study quality, allowing for a ranking based on the total score. More than 80 percent of the total score is considered high quality, between 50 and 80 percent is considered medium quality and less than 50 percent is considered low quality. This systematic assessment method ensures the reliability and scientific rigor of the review's findings.

## 3 Results

**Fig. 1:**
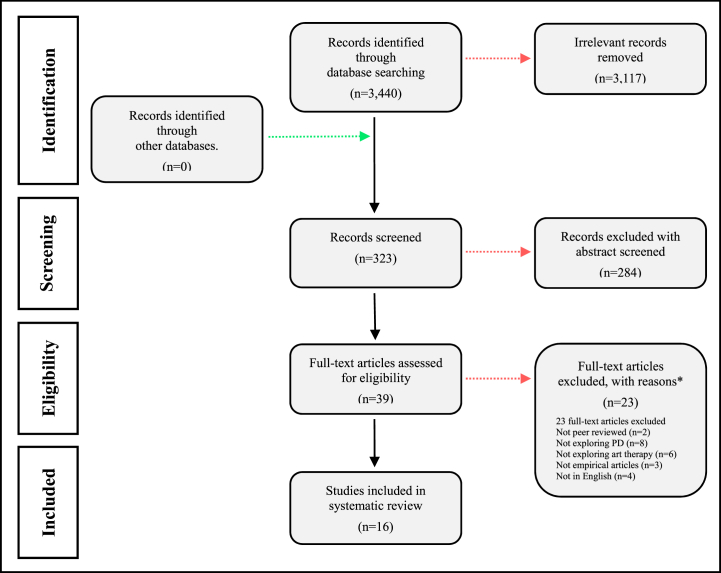
Flow diagram for the included and excluded articles. *Note*. This figure follows by PRISMA-P format = Preferred Reporting Items for Systematic Reviews and Meta-Analyses Protocols. Adapted from Moher et al. (2009). Copyright by 2009 Moher et al. *Articles on art therapy, Parkinson's disease were evaluated in turn.

**Fig. 2:**
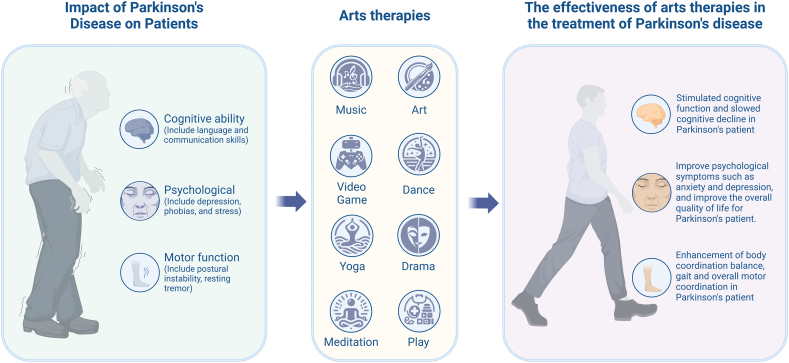
Diagram of the relationship between arts therapies and Parkinson's disease.

The framework shown in Fig. 1 outlines the inclusion and exclusion criteria for the study [25]. In the course of our systematic database search, a total of 3440 records were initially identified. Following the removal of duplicates and the exclusion of records with irrelevant themes, a corpus of 323 articles pertinent to the topic was established, and their abstracts were subsequently subjected to an eligibility assessment. We excluded 284 articles based on an assessment of their abstracts. The remaining 39 articles, encompassing a diverse array of clinical designs, including reviews, clinical studies, case studies, and self-reports, underwent a full-text evaluation. Of these, 23 articles were deemed not to meet the inclusion criteria and were consequently excluded. Ultimately, 16 articles were incorporated into our review. The selection process of the cited articles is delineated in Fig. 1, which illustrates the characteristics and outcomes of the selected studies on arts therapies in the rehabilitation of patients with PD (see Fig. 2).

Table 2 synthesizes the key data from the included studies, and the grid summarizes the information relevant to the objectives of the review.

**Table 2:** Citation analysis and records of full-text review.

References, Country/Region	Types of Art	Types of Study	Study Objective	Participants	Methodology	Analysis	Results
Bega et al. (2017) [26] American	Psychodrama	Randomized Clinical Trial	Develop and study a novel community Improvisation Theater program for PD in order to improve quality of life.	22 subjects were randomized 1:1 to active-start or control-start groups, controlling for age and Hoehn and Yahr stage. Participants were recruited from the Northwestern PD and Movement Disorders Center.	One hour improvisation theater sessions led by The Second City faculty took place once a week for 12 weeks. Each assessment included the Unified Parkinson's Disease Rating Scale (UPDRS) parts I-IV, the PD 39-item Questionnaire (PDQ-39), as well as neurology QOL (neuro-QOL) item bank v 1.0 (short forms) assessment tools of communication, anxiety, stigma, depression, and positive affect and well-being.	Groups are compared at baseline using Fisher's Exact test or Wilcoxon rank sum tests to evaluate equality of groups by randomization group.	A novel improvisation program can be well-attended, enjoyable, and improve Activities of Daily Living measures among patients with PD of varying ages and disease severity.
Fogg-Rogers et al. (2016) [27] United kingdom	Music Therapy	Qualitative Descriptive Research	To explore the experiences of and factors influencing participation in choral singing therapy by people with stroke or Parkinson's disease and their significant others.	Eight had a stroke (four male); Six had Parkinson's disease (four male)	Semi-structured interviews with patients using supported communication methods as needed.	Excerpts of conversations were analyzed inductively in the same way using QSR International NVivo version 9 software (QSR International, Melbourne, Australia) and full verbatim transcripts.	Choral singing was perceived by people with stroke and Parkinson disease to help them self-manage some of the consequences of their condition, including social isolation, low mood and communication difficulties.
Hasan et al. (2022) [28] Egypt	Dance Therapy	Pooled Analysis	To assess the effect of dance in patients with Parkinson's disease.	Fourteen randomized controlled trials with 372 patients were included.	Authors searched PubMed, Scopus, Web of Science, and Cochrane Central Register of Controlled Trials (CENTRAL) till May 2019 and updated our search on April 2020 using the following search strategy: (dance OR dancing OR movement therapy) AND (Parkinson OR Parkinson*).	Data were extracted and pooled as mean difference (MD) with 95 % confidence interval (CI) by Review Manager 5.3.	In comparison to other types of exercise or no activity, dance improves the symptoms and outcomes in patients with PD, especially motor symptoms. Dance also has positive effects on balance, functional mobility, and cognition.
Mitarnun et al. (2022) [29] Thailand	Meditation	Randomized Controlled Trial	To determine the effects of walking meditation on functional performance, disease severity, and anxiety in Parkinson's disease.	Thirty-three participants with PD were randomly allocated to the control group (n = 16) or the walking meditation group (n = 17).	Participants in the walking meditation group were asked to perform WM monthly under supervision and encouraged to practice at home at least 3 days/week for 12 weeks. The measuring tools are Gait velocity, Timed Up and Go, five times sit to stand (FTSTS) test, Unified Parkinson's Disease Rating Scale (UPDRS), and the percentage of participants with anxiety (Hospital Anxiety and Depression Scale-part anxiety [HADS-A] ≥8).	All statistical analyses were performed using Review Manager (version 5.3; Cochrane Collaboration). The mean difference or standardized mean difference was determined as the effect size for continuous outcomes. Outcome accuracy was determined using 95 % confidence intervals with p < 0.05 considered significant. Authors used the DerSimonian and Laird random-effects model to determine a pooled estimate of the mean difference.	Home-based walking meditation can encourage high rates of exercise adherence, reduce disease severity, lower the percentage of participants with anxiety, and might be suitable during disease endemic and/or pandemic in PD.
Kwok et al. (2019) [30] Hong Kong	Yoga	Randomized Clinical Trial	To compare the effects of a mindfulness yoga program vs stretching and resistance training exercise on psychological distress, physical health, spiritual well-being, and health-related quality of life in patients with mild-to-moderate PD.	The 138 participants included 65 men (47.1 %) with a mean age of 63.7 (8.7) years and a mean MDS-UPDRS score of 33.3 (15.3). Participants were randomized 1:1 to mindfulness yoga or stretching and resistance training exercise.	Authors using the validated Hospital Anxiety and Depression Scale (HADS) (Chinese-Cantonese language), validated Movement Disorders Society Unified Parkinson's Disease Rating Scale (MDS-UPDRS), Timed Up and Go Test, validated Holistic Well-being Scale (Chinese version) and validated disease-specific 8-item Parkinson's Disease Questionnaire (Chinese version) to assess patients with PD.	Descriptive statistics were used to summarize the demographics, health conditions, and clinical outcomes of the participants at each time point. The normality of variables was assessed using the skewness statistic and normal probability plot.	Among patients with mild-to-moderate PD, the mindfulness yoga program was found to be as effective as stretching and resistance training exercise in improving motor dysfunction and mobility, with the additional benefits of a reduction in anxiety and depressive symptoms and an increase in spiritual well-being and Health-related quality of life.
Marotta et al. (2022) [31] Italy	Video Game	Systematic Reiew	To explore the present systematic review of randomized controlled trials aimed at assessing the effects of VR and exergames/telerehabilitation in the cognitive rehabilitation management of patients with PD.	N/A	PubMed, Scopus and Web of Science databases were systematically searched up to February 14th, 2022, to identify RCTs assessing patients with PD undergoing cognitive rehabilitation including VR or exergames/telerehabilitation.	The quality assessment was performed following the Version 2 of the Cochrane risk-of-bias tool for randomized trials (RoB 2).	The systematic review showed a positive impact of exergames and VR on cognitive impairment in PD patients. Thus, these two innovative and technological interventions might be part of the complex rehabilitative treatment framework of PD patients.
Tunur et al. (2020) [32] American	Dance Therapy and Video Game	Single-Group Feasibility Pilot Study	To evaluate the feasibility, safety, and acceptability of a mobile dance intervention and obtain preliminary efficacy estimates for assessment of the research protocol.	Seven participants with Parkinson's disease.	Participants with PD were asked to use Google Glass preloaded with ‘Moving Through Dance’ modules for three weeks. Changes in motor functions (balance, mobility) and non-motor functions (mood, quality of life) were evaluated before and after completion of the intervention.	Descriptive statistics were used to profile the study participants and report recruitment, safety, adherence, ‘Moving Through Glass’ usage, and survey outcomes.	The intervention was safe and accepted by participants. Use of ‘Moving Through Glass’ improved mobility with a cognitive load.
Pereira et al. (2019) [33] Brazil	Music Therapy and Dance Therapy	A Review of Evidence	This review aims to demonstrate the efficiency of music and dance for gait improvement and symptom alleviation in Parkinson disease.	N/A	Studies that analyzed sound stimuli and dance in gait improvement in Parkinson disease were searched through PubMed, Scopus, Doaj, MEDLINE, and ScienceDirect databases from November 2017 to April 2018 and repeated in September 2018.	Extract the content and results from the other references and organize the discussion of the efficiency of music and dance for gait improvement and symptom alleviation in Parkinson disease.	Dance and music therapy interventions are noninvasive, simple treatment options, which promote gait and cognition.
Kalyani et al. (2019) [34] Australia	Dance Therapy	Parallel Group Pretest Posttest Study	To determine whether dance classes based on Dance for Parkinson's disease, improve cognition, psychological symptoms and Quality of Life in Parkinson's disease.	17 participants were assigned to the dance group, and 16 participants were assigned to the control group.Participants had early-stage PD (Hoehn & Yahr: DG = 1.6 ± 0.7, CG = 1.5 ± 0.8) with no cognitive impairment (Addenbrooke's score: DG = 93.2 ± 3.6, CG = 92.6 ± 4.3).	The dance group undertook a 1-h class, twice weekly for 12 weeks, while the control group had treatment as usual. Both groups were assessed for disease severity (MDS-UPDRS), cognition (NIH Toolbox® cognition battery, Trail Making Test), psychological symptoms (Hospital Anxiety and Depression Scale, MDS-UPDRS-I) and Quality of Life (PDQ-39, MDS-UPDRS-II).	Demographic data collection and assessments of disease severity and cognitive function were conducted at baseline. Assessments of speech, voice, respiratory function, and voice-related quality of life were conducted at baseline and 3 months by trained assessors.	Group comparison of pre-post change scores showed that selected cognitive skills (executive function and episodic memory), psychological symptoms (anxiety and depression) as well as quality of life were significantly improved by the intervention.
Bae et al. (2018) [35] South Korea	Art Therapy	Observational Study	Investigates how clay art therapy affects Parkinson's disease patients' overall mental and physical condition as well as future treatment potentiality.	A total of 54 patients with Parkinson's disease (control = 28, experimental = 26).	The experimental group received two 80-min clay art therapy sessions per week. The experimental group was introduced to clay art plus usual rehabilitation exercises whereas the control group only maintained routine rehabilitation programs, such as physical activities, game playing, oral-motor exercises, and solving logic/arithmetic puzzles. Outcome measures were assessed at baseline and at 8 weeks, after completion of the program: The Box and Block Test (BBT), scale for self-expression, Geriatric Depression Scale Short Form-Korean Version (GDSSF-K), Parkinson's Disease Quality Of Life (PDQOL).	The data were analyzed using SPSS version 22.0 (IBM Corp, Armonk, NY). Descriptive statistics were used to summarize the participants' demographic and clinical characteristics. In order to determine the homogeneity of the experimental and control group, Chi-square tests and *t*-test were used. To examine the differences between the group variables, *t*-test, analyses of variance, and Scheffé tests were performed.	Study results indicate that clay art is a viable platform of therapy for PD patients. The study findings revealed statistically significant: improved hand dexterity, better mood, elevated self-expression, and enhanced quality of life. Creating art can help distract PD patients from thoughts of the disease, and stimulating a relaxed, peaceful state of mind.
Ban et al. (2021) [36] China	Yoga	Meta-Analysis	To explore whether yoga is beneficial for people with Parkinson's disease and the ways in which yoga can improve their symptoms and enhance their quality of life.	A total of 62 adolescents finally completed the experiment.	A meta-analysis was conducted by systematically searching PubMed, Embase, and Cochrane Library databases till August 2020 for studies published in English. The reference lists of eligible studies were also searched. The motor symptoms (UPDRS-Part III), balance function (BBS and BESTest), functional mobility (TUG), anxiety (HADS and BAI), depression (HADS and BDI), and the quality of life (PDQ-39 and PDQ-8) were the primary evaluation indexes.	For continuous data, the mean differences (MDs) or standardized mean differences (SMDs), with 95 % confidence intervals (CIs), were calculated using RevMan 5.4. If the mean or standard deviation of the changes was not provided, then Cochrane software system was used for the calculation of MD or SMD.	The pooled results showed the benefits of yoga in improving motor function, balance, functional mobility, reducing anxiety and depression, and increasing Quality of Life in PD patients.
Santos et al. (2019) [37] Brazil	Play Therapy	Randomized Clinical Trial	To investigate if the effects of the combination of Nintendo Wii to Conventional Exercises are superior to isolated techniques in the rehabilitation of balance, gait, functional mobility and improvement of the quality of life of individuals with PD.	45 patients with PD were divided into three groups, Nintendo Wii alone, Conventional Exercises alone and Nintendo Wii plus Conventional Exercises.	Interventions were conducted in all groups for 50 min a day, twice a week for eight weeks, accounting for the total of 16 sessions. Patients with PD will be assessed by the Berg Balance Scale (BBS), Dynamic Gait Index (DGI) scale, Timed “Up & Go” test (TUG) and Parkinson's 228 Disease Questionnaire (PDQ-39).	Data was analyzed using statistical software R 3.5.2 (Team, 2013). Descriptive analysis (mean, standard deviation, median and interquartile range for quantitative variables, and absolute and relative frequency for qualitative variables) were performed to identify the characteristics of the study sample.	This study demonstrated that the combination of Nintendo Wii with Conventional Exercises, as well as each single intervention, with the same therapeutic dose, promoted improvement of balance, gait, functional mobility and Quality of Life of patients with PD.
Lin et al. (2023) [38] Taiwan	Meditation	Review and Meta-Analysis	Analysing the impact of investigating mindfulness and meditation therapies on people with PD.	N/A	A literature search was conducted using PubMed, Embase, Cochrane Library, and ClinicalTrials.gov for randomized controlled trials comparing mindfulness and meditation therapies with control treatments in patients with PD.	All statistical analyses were performed using Review Manager (version 5.3; Cochrane Collaboration). The mean difference or standardized mean difference was determined as the effect size for continuous outcomes. Outcome accuracy was determined using 95 % confidence intervals with p < 0.05 considered significant. Authors used the DerSimonian and Laird random-effects model to determine a pooled estimate of the mean difference.	Mindfulness and meditation therapies significantly improved Unified Parkinson's Disease Rating Scale-Part III score and cognitive function. However, no significant differences were discovered between mindfulness therapies and control in gait velocity,activities of daily living, depression, anxiety, pain, or sleep disturbance.
Cucca et al. (2021) [39] American	Art Therapy	Open-label, Prospective, Exploratory Trial	To explore the potential rehabilitative effect of art therapy and its underlying mechanisms in Parkinson's disease.	Observational study of eighteen patients with PD, followed in a prospective, open-label, exploratory trial.	Before and after twenty sessions of art therapy, PD patients were assessed with the UPDRS, Pegboard Test, Timed Up and Go Test (TUG), Beck Depression Inventory (BDI), Modified Fatigue Impact Scale and PROMIS-Self-Efficacy, Montreal Cognitive Assessment, Rey-Osterrieth Complex Figure Test (RCFT), Benton Visual Recognition Test (BVRT), Navon Test, Visual Search, and Stop Signal Task. Eye movements were recorded during the BVRT. Resting-state functional MRI (rs-fMRI) was also performed to assess functional connectivity (FC) changes within the dorsal attention (DAN), executive control (ECN), fronto-occipital (FOC), salience (SAL), primary and secondary visual (V1, V2) brain networks. Authors also tested fourteen age-matched healthy controls at baseline.	Partial correlations were performed, with age and gender as covariates, between clinical, visuospatial and functional connectivity outcomes in both PD and control groups together, and in each group separately (PD-baseline, PD-follow-up, and controls). A bivariate regression model was applied to account for age and gender variability. Variables showing a Pearson coefficient ≥ ±0.75 were considered strongly correlated.	Art therapy improves overall visual-cognitive skills and visual exploration strategies as well as general motor function in patients with PD. The changes in brain connectivity highlight a functional reorganization of visual networks.
Pohl et al. (2013) [40] Sweden	Music Therapy	Randomized Clinical Trial	To assess the feasibility of the novel intervention, Ronnie Gardiner Rhythm and Music (RGRM™) Method compared to a control group for patients with Parkinson's disease.	18 patients, mean age 68 years. There were 12 people in the intervention group and 6 people in the control group.	Feasibility was assessed by comparing effects of the intervention on clinical outcome measures. The primary outcome was mobility as measured by a computer-based, two-dimensional motion analysis system based on the Posturo-LocomotionManual (PLM) method, developed for patients with PD. Secondary outcomes were two further measures for mobility (Timed-Up-and-Go (TUG), and UPDRS motor score), quality of life using t he Swedish version of the Parkinso Disease Questionnaire-39 summary index (PDQ -39), and six measures for cognitive ability. This was evaluated with the Cognitive Assessment battery (CAB).	Data are presented as mean (SD) or median (IQR). The Mann -Whitney *U* test was used to test differences between groups and χ2 or Fisher's exact test was used for differences in proportions. Comparisons within groups were analyzed using Wilcoxon signed rank test. No adjustments for multiple comparisons were made. A p value of 0.05 was considered to be statistically significant. All analysis was performed using SPSS 17.0 (SPSS Inc., Chicago, IL, USA). For power analysis the “Russ Lenth's power and sample size page” was used.	The RGRM™ Method appeared to be a useful and safe method that showed promising results in both motor and cognitive functions as well as quality of life in patients with moderate Parkinson's disease.
Dauvergne et al. (2018) [41] France	Music Therapy and Vedio Game	Prospective Randomized Study	To evaluate the adherence, usability and acceptance of a rehabilitation protocol with a music-based serious game and its effect on rhythmic skills in Parkinson disease.	The study population consisted of 16 Parkinson's disease patients with mild cognitive and motor deficits.	Rehabilitation consisted of a 6-week at-home training program targeting rhythmic skills with a dedicated SG, Rhythm Workers, implemented on a tablet device. Patients were asked to play the game at least 30 min, 3 times a week. Two half-day evaluations were conducted before and after rehabilitation. Time played and average game scores were recorded. Suitability was evaluated by a questionnaire inspired by the Suitability Evaluation Questionnaire (SEQ) and rhythmic skills by the Beat Alignment Test from the Battery for the Assessment of Auditory Sensorimotor and Timing Abilities (BAASTA).	Expanded and analyzed the data (Suitability Evaluation Questionnaire，Beat Alignment Test，Time played，Mean score in Rhythm Workers and game achievement) in both tabular and textual form.	This study showed good to excellent suitability of an serious game used on a tablet interface for rhythmic training in Parkinson's disease and the feasibility of this type of training in this population.

**Abbreviation:** PD= Parkinson disease; N/A means no participants.

The included studies provided accurate data and study content. The quality of the included studies was assessed by the researchers through the use of the JBI Critical Appraisal Inventory, and the quality of the included studies was relatively high (Table 3).

**Table 3:** Summary of the quality of citation score.

Authors	Study Design	JBI Critical Assessment Tool	Assessment of the quality of the study													Overall Score
			1	2	3	4	5	6	7	8	9	10	11	12	13	
Fogg-Rogers et al. (2016) [27]	Qualitative Descriptive Research	JBI Checklist for Qualitative Research	Y	Y	Y	Y	Y	N	Y	N	Y	Y				High quality(8/10)
(Pohl et al., 2013) [40]	Randomized Controlled Trial	JBI Critical Appraisal Checklist for Randomized Controlled Trials	Y	Y	UN	N	UN	UN	Y	Y	Y	UN	UN	Y	Y	Medium quality(7/13)
(Bega et al., 2017) [26]	Randomized Controlled Trial	JBI Critical Appraisal Checklist for Randomized Controlled Trials	Y	N	UN	Y	Y	N	UN	Y	Y	UN	UN	Y	Y	Medium quality(7/13)
(Santos et al., 2019) [37]	Randomized Controlled Trial	JBI Critical Appraisal Checklist for Randomized Controlled Trials	Y	UN	Y	UN	N	N	Y	Y	Y	UN	Y	Y	Y	Medium quality(8/13)
(Mitarnun, 2022) [29]	Randomized Controlled Trial	JBI Critical Appraisal Checklist for Randomized Controlled Trials	Y	UN	UN	UN	Y	Y	UN	Y	Y	Y	Y	Y	Y	Medium quality(9/13)
(Kwok et al., 2019) [30]	Randomized Controlled Trial	JBI Critical Appraisal Checklist for Randomized Controlled Trials	Y	Y	Y	UN	N	Y	Y	Y	Y	Y	Y	Y	Y	Hhigh quality(11/13)
Lin et al. (2023) [38]	Review and Meta-Analysis	JBI Checklist for Systematic Reviews and Research Syntheses	Y	Y	Y	Y	Y	Y	Y	UN	Y	Y	Y			High quality(10/11)
Hasan et al. (2022) [28]	Pooled Analysis	JBI Checklist for Systematic Reviews and Research Syntheses	Y	UN	Y	N	Y	UN	Y	UN	Y	Y	Y			Medium quality(7/11)
Marotta et al. (2022) [31]	Systematic Review	JBI Checklist for Systematic Reviews and Research Syntheses	Y	Y	Y	Y	Y	Y	UN	UN	Y	Y	Y			High quality(9/11)
Ban et al. (2021) [36]	Meta-Analysis	JBI Checklist for Systematic Reviews and Research Syntheses	Y	Y	UN	Y	Y	Y	UN	UN	Y	Y	Y			Medium quality(8/11)
Pereira et al. (2019) [33]	A Review of Evidence	JBI Checklist for Systematic Reviews and Research Syntheses	Y	Y	UN	Y	UN	UN	UN	UN	UN	Y	Y			Low quality(5/11)
Kalyani et al. (2019) [34]	Parallel Group Pretest Posttest Study	JBI Checklist for Quasi-Experimental Studies	Y	N	Y	UN	Y	Y	Y	UN	Y					Medium quality(7/9)
Cucca et al. (2021) [39]	Open-label, Prospective, Exploratory Trial	JBI Checklist for Quasi-Experimental Studies	Y	Y	UN	UN	Y	Y	Y	UN	Y					Medium quality(6/9)
Tunur et al. (2020) [32]	Single-Group Feasibility Pilot Study	JBI Checklist for Quasi-Experimental Studies	Y	N	UN	UN	Y	Y	Y	UN	Y					Medium quality(5/9)
Dauvergne et al. (2018) [41]	Prospective Randomized Study	JBI Checklist for Quasi-Experimental Studies	Y	N	N	N	Y	Y	Y	UN	Y					Medium quality(5/9)
Bae et al. (2018) [35]	Observational Study	JBI Checklist for Case Control Studies	UN	Y	Y	Y	Y	UN	UN	Y	UN	Y				Medium quality(6/11)

Abbreviations: JBI, the Joanna Briggs Institute; Y, Yes; N, No; UN, Unclear.

JBI Checklist for Case Control Studies: 1.Were the groups comparable other than the presence of disease in cases or the absence of disease in controls? 2.Were cases and controls matched appropriately? 3.Were the same criteria used for identification of cases and controls? 4.Was exposure measured in a standard, valid and reliable way? 5.Was exposure measured in the same way for cases and controls? 6.Were confounding factors identified? 7.Were strategies to deal with confounding factors stated? 8.Were outcomes assessed in a standard, valid and reliable way for cases and controls? 9.Was the exposure period of interest long enough to be meaningful? 10.Was appropriate statistical analysis used?.

JBI Critical Appraisal Checklist for Randomized Controlled Trials: 1.Was true randomization used for assignment of participants to treatment groups? 2.Was allocation to treatment groups concealed? 3.Were treatment groups similar at the baseline? 4.Were participants blind to treatment assignment? 5.Were those delivering the treatment blind to treatment assignment? 6.Were treatment groups treated identically other than the intervention of interest? 7.Were outcome assessors blind to treatment assignment? 8.Were outcomes measured in the same way for treatment groups? 9.Were outcomes measured in a reliable way 10.Was follow up complete and if not, were differences between groups in terms of their follow up adequately described and analyzed? 11.Were participants analyzed in the groups to which they were randomized? 12.Was appropriate statistical analysis used? 13.Was the trial design appropriate and any deviations from the standard RCT design (individual randomization, parallel groups) accounted for in the conduct and analysis of the trial?.

JBI checklist for Systematic Reviews and Research Syntheses:1. Is the review question clearly and explicitly stated? 2.Were the inclusion criteria appropriate for the review question? 3.Was the search strategy appropriate? 4.Were the sources and resources used to search for studies adequate? 5.Were the criteria for appraising studies appropriate? 6.Was critical appraisal conducted by two or more reviewers independently? 7.Were there methods to minimize errors in data extraction? 8.Were the methods used to combine studies appropriate? 9.Was the likelihood of publication bias assessed? 10.Were recommendations for policy and/or practice supported by the reported data? 11.Were the specific directives for new research appropriate?.

JBI Checklist for Quasi-Experimental Studies: 1.Is it clear in the study what is the “cause” and what is the “effect” (i.e. there is no confusion about which variable comes first)? 2.Was there a control group? 3.Were participants included in any comparisons similar? 4.Were the participants included in any comparisons receiving similar treatment/care, other than the exposure or intervention of interest? 5.Were there multiple measurements of the outcome, both pre and post the intervention/exposure? 6.Were the outcomes of participants included in any comparisons measured in the same way? 7.Were outcomes measured in a reliable way? 8.Was follow-up complete and if not, were differences between groups in terms of their follow-up adequately described and analyzed? 9.Was appropriate statistical analysis used?.

JBI Checklist for Qualitative Research: 1.Is there congruity between the stated philosophical perspective and the research methodology? 2.Is there congruity between the research methodology and the research question or objectives? 3.Is there congruity between the research methodology and the methods used to collect data? 4.Is there congruity between the research methodology and the representation and analysis of data? 5.Is there congruity between the research methodology and the interpretation of results? 6.Is there a statement locating the researcher culturally or theoretically? 7.Is the influence of the researcher on the research, and vice-versa, addressed? 8.Are participants, and their voices, adequately represented? 9.Is the research ethical according to current criteria or, for recent studies, and is there evidence of ethical approval by an appropriate body? 10.Do the conclusions drawn in the research report flow from the analysis, or interpretation, of the data?.

In addition, the researchers used the PICO(s) study design to identify RCTs data from these 16 studies (Table 4).

**Table 4:** Principal characteristics of all included RCTs in this review.

Reference	ID	Types	Sample Size (T/C)	Treatment group		Outcome Measurement Methods	Control Group
				Intervention	Procedure Times		
(Pohl et al., 2013) [40]	31(1)	RCT	12/6	MT	12	PLM/TUG/UPDRS/PDQ-39/CAB	TT
(Bega et al., 2017) [26]	33(2)	RCT	11/11	PA	12	UPDRS/PDQ -39/QOL	CS
(Santos et al., 2019) [37]	37(3)	RCT	13/14/14^a^	PT	8	BBS/DGI/TUG/PDQ-39	TT
(Mitarnun, 2022) [29]	43(4)	RCT	17/16	WM	36	FTSTS/UPDRS/HADS-A	TT
(Kwok et al., 2019) [30]	44(5)	RCT	71/67	YT	8	HADS/MDS-UPDRS/TUG/HWS/PDQ-8/HRQOL	TT

Abbreviations: MT = Music Therapy; TT = Traditional therapy; PA=Psychodrama; Play Therapy; CS = Controlstart; DT = Dance Therapy; WM = Walking Meditation; YT = Yoga Therapy; PLM = Posturo-LocomotionManual; TUG = Timed-Up-and-Go, UPDRS = Unified Parkinson's Disease Rating Scale; PDQ-39 = Parkinso Disease Questionnaire-39 summary index; CAB = Cognitive Assessment battery; PD 39-item Questionnaire (PDQ-39); QOL = quality of life; BBS = Berg Balance Scale; DGI = Dynamic Gait Index; FTSTS = Five Times Sit To Stand; HADS-A = Hospital Anxiety and Depression Scale-part anxiety; HADS = Hospital Anxiety and Depression Scale; MDS-UPDRS = Movement Disorders Society Unified Parkinson's Disease Rating Scale; HWS = Holistic Well-being Scale; PDQ-8 = Parkinso Disease Questionnaire-8 summary index; HRQOL = Health-related quality of life.

a ID-37(3) group has split into 3 different intervention therapy which is N = 13/14/14, the whole count number is 41.

### 3.1 Arts therapies method for Parkinson's patients

Patients with PD commonly experience a wide range of physical and psychological challenges, which are attributed to the complex nature of this neurodegenerative disorder [[42], [43], [44], [45], [46], [47], [48]]. Unlike traditional therapeutic interventions that mainly target symptomatic relief [49,50], arts therapies offer a distinctive rehabilitative strategy [[51], [52], [53]]. This approach provides innovative forms and methods for recovery, leveraging non-verbal communication and creativity to aid individuals with PD [54,55]. Arts therapies encompass various modalities, making it a multifaceted treatment option due to its diverse types [56]. This form of therapy includes music therapy [[57], [58], [59]], meditation therapy [[60], [61], [62]], yoga therapy [63,64], art therapy [[65], [66], [67]], dance therapy [21,[68], [69], [70]], drama therapy [[71], [72], [73]], video game therapy [[74], [75], [76]], play therapy [[77], [78], [79]], and color therapy [80,81].

As shown in Table 2, the literature included in this study contained most of the types of arts therapies, such as meditation, dance therapy, art therapy, yoga, music therapy, video game therapy, drama therapy, and play therapy. However, no studies related to color therapy were found in this inclusion.

### 3.2 Therapeutic summary of arts therapies

Arts therapies, encompassing a range of non-pharmacological interventions including art therapy, meditation, dance therapy, yoga, music therapy, play therapy, and psychodrama, have demonstrated significant efficacy in ameliorating both the physical and psychological symptoms associated with PD [28,29,31,33,36,39,41]. These therapeutic approaches effectively address the complex challenges encountered by individuals with PD. They contribute to enhancing motor function, mood elevation, anxiety reduction, cognitive enhancement, and overall quality of life improvement [34,37,40]. This underscores the value of arts therapies in the comprehensive management of PD symptoms and the enhancement of patient well-being. The paper presents a tabular summary that clearly delineates the outcomes from eight distinct therapeutic approaches, as documented in existing literature (Table 5).

**Table 5:** Comparative analysis of the results of different methods.

Methods	Author	Types of study	Results
Art Therapy	Bae et al. (2018) [35]	Observational Study	Study results indicate that clay art is a viable platform of therapy for parkinson disease patients. The study findings revealed statistically significant: improved hand dexterity, better mood, elevated self-expression, and enhanced quality of life. Creating art can help distract parkinson disease patients from thoughts of the disease, and stimulating a relaxed, peaceful state of mind.
	Cucca et al. (2021) [39]	Open-label, Prospective, Exploratory Trial	Art therapy improves overall visual-cognitive skills and visual exploration strategies as well as general motor function in patients with parkinson disease. The changes in brain connectivity highlight a functional reorganization of visual networks.
Meditation	Mitarnun et al. (2022) [29]	Randomized Controlled Trial	Home-based walking meditation can encourage high rates of exercise adherence, reduce disease severity, lower the percentage of participants with anxiety, and might be suitable during disease endemic and/or pandemic in parkinson disease.
	Lin et al. (2023) [38]	Review and Meta-Analysis	Mindfulness and meditation therapies significantly improved Unified Parkinson's Disease Rating Scale-Part III score and cognitive function. However, no significant differences were discovered between mindfulness therapies and control in gait velocity,activities of daily living, depression, anxiety, pain, or sleep disturbance.
Dance Therapy	Hasan et al. (2022) [28]	Pooled Analysis	In comparison to other types of exercise or no activity, dance improves the symptoms and outcomes in patients with parkinson disease, especially motor symptoms. Dance also has positive effects on balance, functional mobility, and cognition.
	Kalyani et al. (2019) [34]	Parallel Group Pretest Posttest Study	Group comparison of pre-post change scores showed that selected cognitive skills (executive function and episodic memory), psychological symptoms (anxiety and depression) as well as quality of life were significantly improved by the intervention.
Yoga	Kwok et al. (2019) [30]	Randomized Controlled Trial	Among patients with mild-to-moderate parkinson disease, the mindfulness yoga program was found to be effective in improving motor dysfunction and mobility, with the additional benefits of a reduction in anxiety and depressive symptoms and an increase in spiritual well-being and health-related quality of life.
	Ban et al. (2021) [36]	Meta-Analysis	The pooled results showed the benefits of yoga in improving motor function, balance, functional mobility, reducing anxiety and depression, and increasing quality of life in parkinson disease patients.
Music Therapy	Fogg-Rogers et al. (2016) [27]	Qualitative Descriptive Research	Choral singing was perceived by people with stroke and Parkinson disease to help them self-manage some of the consequences of their condition, including social isolation, low mood and communication difficulties.
	Pohl et al. (2013) [40]	Randomized Clinical Trial	The RGRM™ Method appeared to be a useful and safe method that showed promising results in both motor and cognitive functions as well as quality of life in patients with moderate Parkinson's disease.
Play Therapy	Santos et al. (2019) [37]	Randomized Clinical Trial	This study demonstrated that the combination of Nintendo Wii with Conventional Exercises, as well as each single intervention, with the same therapeutic dose, promoted improvement of balance, gait, functional mobility and Quality of life of patients with parkinson disease.
Video Game	Marotta et al. (2022) [31]	Systematic Review	Exergaming and VR might be considered promising rehabilitation interventions in the cognitive rehabilitation framework of parkinson disease patients, it improves patients' cognitive and executive functions.
Psychodrama	Bega, D. et al. (2017) [26]	Randomized Clinical Trial	A novel improvisation program can be well-attended, enjoyable, and improve activities of daily living measures among patients with parkinson disease of varying ages and disease severity.
Dance Therapy and Video Game	Tunur et al. (2020) [32]	Single-Group Feasibility Pilot Study	The intervention was safe and accepted by participants. Use of ‘Moving Through Glass’ improved mobility with a cognitive load.
Music Therapy and Dance Therapy	Pereira et al. (2019) [33]	A Review of Evidence	Dance and music therapy interventions are noninvasive, simple treatment options, which promote gait and cognition in PD patient.
Music Therapy and Vedio Game	Dauvergne et al. (2018) [41]	Prospective Randomized Study	This study showed good to excellent suitability of an serious game used on a tablet interface for rhythmic training in Parkinson's disease and the feasibility of this type of training in this population.

Arts therapies have emerged as a promising therapeutic modality in the rehabilitation of PD patients, incorporating a diverse array of artistic activities such as oil painting, clay sculpture, decorative textiles, and general painting. These therapies have been shown to enhance impaired visuospatial functions, including visually directed attention, shape recognition, motion perception, abstraction, sensorimotor integration, and hand-eye coordination [39]. Additionally, the creative process involved in producing art offers psychological benefits by distracting patients from their symptoms, promoting relaxation and calmness, and thereby improving self-awareness, mood, and reducing anxiety [35].

Psychodrama, particularly through initiatives like Bega's Community Improvisation Theatre Project designed for PD patients, fosters social interaction and support within a community setting, which is fundamental in aiding relaxation and mood enhancement [26]. Music therapy, including choir singing therapy and the Ronald Gardner Rhythm and Music approach, plays a pivotal role in symptom management, motor and cognitive function enhancement, and elevating quality of life by utilizing musical rhythms as external cues [27,40]. Moreover, music therapy can be combined with serious games to improve the rhythmic skills of PD patients by utilizing melodic and rhythmic elements synchronized with motor tasks [41].

Dance therapy, utilizing the rhythmic aspects of music coupled with physical movement, offers a comprehensive approach to PD rehabilitation, improving coordination, balance, gait, and motor control [28,33]. It also positively impacts psychological symptoms and cognitive functions, such as executive functioning and situational memory [34]. In addition, innovative methods like augmented reality and Google Glass-based dance techniques further demonstrate the efficacy of video game-based interventions in enhancing flexibility in physical activities and cognitive load in PD patients [32]. Besides, virtual reality-based exercises, like those involving the Nintendo Wii, have been identified as effective for PD rehabilitation [37]. Exergames and virtual reality have a positive impact on cognitive impairment in patients with PD [31].

Yoga has been recognized for its substantial benefits in improving motor function, balance, functional flexibility, and reducing anxiety and depression, thereby enhancing mental health and quality of life for PD patients [30,36]. Complementary to yoga, home-based walking meditation has shown high adherence rates and effectiveness in reducing disease severity, anxiety, and improving cognitive performance [29,38]. However, the exploration into the use of color therapy in PD rehabilitation remains insufficient, despite its potential to influence mood and cognitive functions adversely affected in PD [8,48].

In conclusion, arts therapies that incorporate music therapy, dance therapy, yoga therapy, meditation, psychodrama, video games, play therapy, and art therapy provide a comprehensive and multifaceted approach to the rehabilitation of individuals with PD. These arts therapies not only address physical and motor symptoms, but also greatly contribute to the mental and emotional well-being of patients with PD. Furthermore, there is a lack of empirical evidence supporting the efficacy of color therapy in the rehabilitation process of PD.

### 3.3 Literature quality assessment

We carefully assessed the overall quality of each study through the JBI quality assessment methodology (Table 3). The results showed that the five studies assessed using the JBI Critical Appraisal Checklist for Randomized Controlled Trials tool had a mean score of 8.4 and a mean overall score of 64.6 %. The mean score of the five studies evaluated using the JBI Checklist for Systematic Reviews and Research Syntheses tool was 7.8, and the mean overall score was 70.9 %. The four studies assessed using the JBI Checklist for Quasi-Experimental Studies tool had a mean score of 5.75 and an average overall score of 63.8 %. One study assessed using the JBI Checklist for Qualitative Research tool had a mean score of 8, with an average overall score of 80 %. One study assessed using the JBI Checklist for Case Control Studies tool scored 6 with an average overall score of 54.5 %.

Therefore, the study with the highest mean total score in this quality assessment was the one assessed using the JBI Checklist for Qualitative Research tool [27], while the study with the lowest mean score was the one assessed using the JBI Checklist for Case Control Studies tool [35]. However, both of these studies included only one study, and when the average total score of multiple results was considered, the five studies assessed using the JBI Checklist for Systematic Reviews and Research Syntheses tool had the highest average total scores [28,31,33,36,38], and the five studies assessed using the JBI Checklist for Quasi-Experimental Studies tool had the lowest mean total scores [32,34,39,41], and the mean total scores of the five studies assessed using the JBI Critical Appraisal Checklist for Randomized Controlled Trials tool were moderate [26]. Total scores were moderate [26,29,30,37,40]. However, it is worth noting that although the five studies assessed using the JBI Checklist for Systematic Reviews and Research Syntheses tool had the highest average total score, the highest scoring study among them received a score of 10 [38], whereas the lowest scoring study received only a score of 5 [33], with a large difference in quality between the two groups of studies. In contrast, the difference in quality between the five studies assessed using the JBI Critical Appraisal Checklist for Randomized Controlled Trials tool was smaller, so the overall quality of the studies appeared more robust [26,30,40].

Additionally, we conducted an in-depth analysis using the JBI Checklist for Systematic Reviews and Research Syntheses, the JBI Checklist for Quasi-Experimental Studies, and the JBI Critical Appraisal Checklist for Randomized Controlled Trials. In the JBI Checklist for Systematic Reviews and Research Syntheses, questions 1, 10, and 11 received consistent affirmative responses. This consistency underscores a fundamental alignment among the studies regarding the clarity of the review question, the support for recommendations, and the appropriateness of directives for future research. However, question 8 frequently received "No" or "Unclear" responses, highlighting the need for methodological improvements to ensure the reliability and validity of findings. Similarly, in the JBI Checklist for Quasi-Experimental Studies, questions 1, 5, 6, 7, and 9 consistently received affirmative responses, reflecting a clear understanding of causal relationships, robust outcome measurements, and suitable statistical analyses. Conversely, questions 4 and 8 often received "No" or "Unclear" responses, indicating the necessity for better methodological practices, particularly in ensuring comparable treatments and thorough follow-up procedures. Finally, in the JBI Critical Appraisal Checklist for Randomized Controlled Trials, questions 1, 12, and 13 consistently received affirmative responses, demonstrating alignment regarding true randomization, appropriate statistical analyses, and suitable trial design. However, with the exception of Bega's study, question 4 frequently received "No" or "Unclear" responses, emphasizing the need for improvements in participant blinding.

Furthermore, there were a total of four studies that received high scores on this quality scale: those of Fogg-Rogers, Kwok, Lin, and Marotta [27,30,31,38]. These study design types included qualitative descriptive research, systematic review, review and meta-analysis, and randomized controlled trials. The arts therapies that were examined were dance therapy, music therapy, yoga, and meditation. All of these arts therapies have improved symptoms in people with PD.

### 3.4 Therapeutic effects of arts therapies

Table 4 demonstrates the PICO(S) framework, which was used by researchers to extract data from five robust quality RCT studies comprising a total of 252 participants [26,29,30,37,40]. The framework illustrates the use of music therapy, play therapy, yoga therapy, psychodrama, and meditation in addressing people with PD in terms of mental and physical health issues. Results from five RCTs have shown positive outcomes from these therapeutic interventions.

In addition, PICO(s) In an analytical examination of five randomized controlled trials, four studies used the Unified PD Rating Scale to assess the severity of PD patients.Concurrently, three studies used the Timed Up and Go test to measure patients' dynamic balance and gait rhythm. Kwok and colleagues evaluated the overall sense of well-being in PD patients using the Happiness With Scales. Other RCTs applied various scales to assess multiple aspects of PD patients, including disease progression, quality of life, motor impairments, anxiety, depression, balance, and dynamic gait.

These studies effectively evaluated the potential value of various arts therapies methods in improving rehabilitation outcomes for PD patients. Current research demonstrates that arts therapies significantly contribute to the motor, psychological, and cognitive rehabilitation of PD patients [28,32,42]. Consequently, we provide a comprehensive breakdown in Table 6 of the effects that various forms and techniques of arts therapies have on the rehabilitation of individuals suffering from PD (Table 6).

**Table 6:** Rehabilitation effects of different methods.

Methods	Author	Motion Rehabilitation	Psychological Rehabilitation	Cognitive Rehabilitation
Art Therapy	Bae et al. (2018) [35]	**✓**	**✓**	
	Cucca et al. (2021) [39]	**✓**		**✓**
Meditation	Mitarnun et al. (2022) [29]	**✓**	**✓**	
	Lin et al. (2023) [38]			**✓**
Dance Therapy	Hasan et al. (2022) [28]	**✓**		**✓**
	Kalyani et al. (2019) [34]		**✓**	**✓**
Yoga	Kwok et al. (2019) [30]	**✓**	**✓**	
	Ban et al. (2021) [36]	**✓**	**✓**	
Music Therapy	Fogg-Rogers et al. (2016) [27]		**✓**	
	Pohl et al. (2013) [40]	**✓**		**✓**
Play Therapy	Santos et al. (2019) [37]	**✓**		
Video Game	Marotta et al. (2022) [31]			**✓**
Psychodrama	Bega, D. et al. (2017) [26]		**✓**	
Dance Therapy and Video Game	Tunur et al. (2020) [32]			**✓**
Music Therapy and Dance Therapy	Pereira et al. (2019) [33]	**✓**		**✓**
Music Therapy and Vedio Game	Dauvergne et al. (2018) [41]	**✓**		

As shown in Table 6, with the exception of psychodrama, the remainder of the artistic therapies demonstrated considerable efficacy in the motor rehabilitation of PD patients, especially in reducing the symptoms of tremor, rigidity and bradykinesia associated with PD [[28], [29], [30],^33^,[35], [36], [37],[39], [40], [41]]. In addition, art therapy, meditation, dance therapy, yoga, music therapy, and psychodrama can produce a wide range of psychological benefits for PD patients, including emotional, cognitive, and social benefits, and significantly enhancing the mental health of patients with PD [26, 27, 29.30, 34, 35, 36]. Furthermore, music therapy, mediation, dance therapy, art therapy, video game can improve cognitive deficits such as executive functioning and memory difficulties that are often seen in PD patients [28,[31], [32], [33], [34],[38], [39], [40]]. Additionally, arts therapies that incorporate new media technologies, such as video game and play therapy, have proven effective in the motor and cognitive rehabilitation of PD patients [31,37,41]. However, further research is necessary to elucidate the psychological rehabilitation benefits of these new media technology-based therapies for PD patients.

## 4 Discussion

PD is a neurodegenerative disorder marked by the progressive degeneration of dopaminergic neurons in the substantia nigra, necessitating a focus on symptomatic relief in the absence of a definitive cure [48,82]. Pharmacological treatment, particularly through the administration of levodopa in combination with carbidopa, serves as the cornerstone of PD management, enhancing dopamine levels while mitigating peripheral side effects [47,83]. Despite Levodopa's efficacy for PD's motor symptoms, its prolonged use is linked to complications like motor fluctuations and dyskinesias [84]. Additionally, non-motor symptoms, including sleep disturbances, anosmia, constipation, and depression, manifest early and, alongside cognitive decline and dysautonomia, intensify throughout the disease's progression, often dominating its advanced stages [85]. Therefore, the development of safer, non-pharmacological treatments with fewer side effects has become a key area of research in the current treatment of PD.

Arts therapies play a significant role in improving motor skills and physical coordination in PD patients. Activities like painting and working with clay enhance fine motor skills, hand-eye coordination, and flexibility [35,39], while dance and yoga are particularly effective in boosting balance, gait, and overall motor coordination [28,30,36]. Moreover, arts therapies, including meditation, video games, play therapy, and music, provide engaging rehabilitation exercises that offer enjoyment and therapeutic benefits [29,31,37,40]. Beyond physical rehabilitation, arts therapies afford a medium for emotional expression and cognitive engagement, which is crucial for PD patients facing emotional and cognitive challenges [86,87]. These therapies facilitate the expression of emotions and experiences, aiding those with speech difficulties [88,89], and have demonstrated effectiveness in elevating mood, reducing symptoms of depression and anxiety, and enhancing psychological well-being [26,29,30]. The creative processes inherent in arts therapies not only invigorate cognitive function, potentially decelerating cognitive decline, but also heighten patient motivation, thereby improving treatment outcomes. This aspect is vital for PD patients, who might isolate themselves due to motor impairments [90]. Artistic engagement boosts confidence and self-esteem and offers a constructive way to confront disease challenges [35]. Although research increasingly supports arts therapies' potential to improve PD patients' quality of life, the scientific evidence remains less robust than that for traditional treatments. There is a pressing need for further high-quality, large-scale studies to substantiate their efficacy and safety [27,32]. In summary, arts therapies constitute a crucial component of comprehensive PD care. Its capacity to simultaneously address the intricate interplay of motor and non-motor symptoms and augment the efficacy of conventional treatments through creative and appealing methods underscores its importance as a therapeutic modality within the multidisciplinary management of PD [30,91].

Arts therapies constitute a crucial component comprehensive PD care and treatment [14,17]. Its capacity to simultaneously address the intricate interplay of motor and non-motor symptoms and augment the efficacy of conventional treatments through creative and appealing methods underscores its importance as a therapeutic modality within the multidisciplinary management of PD [30,91]. Our review underscores that most arts therapies contribute to motor, psychological, and cognitive rehabilitation, thereby improving PD patients' quality of life. However, yoga therapy has not shown significant effects on cognitive rehabilitation, game therapy on psychological and cognitive rehabilitation, and psychodrama on both motor and cognitive rehabilitation. Future investigations should delve into yoga's impact on cognitive functions in PD patients, explore game therapy for psychological and cognitive recovery further, and examine the effects of psychodrama on motor and cognitive rehabilitation. Additionally, the literature review revealed a gap in research focusing on color therapy and its impact on PD. Patients with PD often suffer from mood disorders such as depression and anxiety [9,46]. Considering that exposure to specific colors can evoke specific emotional responses and influence mood states in patients, color therapy could be used as a supplementary approach in future studies to improve mood regulation and overall state of mind in PD patients [81,[92], [93], [94]].

In addition, Combining new media technologies with arts therapies for PD exhibits considerable promise. This approach combines the traditional benefits of arts therapies with cutting-edge technology, rendering the treatment more personalized, interactive, and broadly applicable. For instance, research by Tunur demonstrated augmented reality's potential when combined with dance to enhance mobility in PD patients with cognitive load [32]. Similarly, studies by Santos and Dauvergne corroborated significant improvements in balance, gait, and cognitive functions through the integration of virtual reality and serious games with traditional exercises [37,41]. These advancements underscore the personalized and participatory aspects of these treatments, contributing to improved outcomes. However, future research is essential to address persisting challenges, including small sample sizes, the absence of control groups, technological barriers, and the high costs associated with devices such as Google Glass. Improving research methodology, overcoming technological barriers, and ensuring the cost-effectiveness of devices are paramount for broadening patient access to these innovative treatments. Additionally, diversifying psychological assessment tools can provide a more comprehensive evaluation of psychological rehabilitation in PD patients.

Moreover, considering the substantial population of PD patients, particularly those with mobility impairments that prevent them from visiting treatment centre, this challenge has become especially pronounced during and following the COVID-19 pandemic [95,96]. Therefore, we propose a hypothesis to deliver arts therapies to PD patients remotely by integrating new media technologies with metaverse techniques. This approach has the potential to revolutionize PD care by enhancing accessibility, personalization, and interactivity. However, navigating the nascent stage of metaverse technology requires addressing data privacy, technological barriers, and cost-effectiveness to ensure safe and effective application in arts therapies for PD patients. Extensive research is essential to evaluate the potential and limitations of metaverse technology in this field comprehensively.

## Limitations

Our review is subject to several limitations that merit acknowledgment. Initially, to ensure that the review team was able to conduct more effective and in-depth analyses within their own language skills, this survey was limited to studies published in English, thus limiting our sample size. As a result, valuable insights from research disseminated in other languages may have been inadvertently overlooked. This limitation may introduce a bias because our findings may not fully reflect or apply to non-English-speaking populations, creating potential study bias.

Additionally, given the small sample size and limited number of studies included in our review, we advocate cautious interpretation of the qualitative results. The constrained sample size means that the conclusions drawn may not be fully representative of broader characteristics, limiting the generality of our findings. Consequently, the insights garnered from our review, while valuable, should be considered preliminary and require further empirical validation across diverse linguistic and cultural contexts to ascertain their general applicability and to mitigate the risk of overlooking significant contributions to the field.

## Conclusions

In conclusion, the existing literature highlights the great potential of arts therapies in the rehabilitation of people with PD, further confirming the efficacy of arts therapies in enhancing the motor, psychological and cognitive rehabilitation process of people with PD. In addition, this review identifies research gaps in the use of color therapy in PD rehabilitation and highlights the need for further exploration of various arts therapies modalities, such as color therapy, in order to realize the full potential of arts therapies in PD treatment.

## Ethics statement

Due to the nature of this study being a literature review and the absence of data collection or analysis, obtaining evaluation and permission from an ethical committee was unnecessary. Informed consent was also not required for this study for the same reason.

## Funding information

This work was supported by the Macau University of Science and Technology's Faculty Research Grant (No.: FRG-24–049-FA)

## Data availability

No data was used for the research described in the article.

## CRediT authorship contribution statement

**Yiyuan Li:** Writing – review & editing, Writing – original draft, Visualization, Validation, Supervision, Software, Resources, Methodology, Investigation, Formal analysis, Data curation, Conceptualization. **Xuexing Luo:** Writing – review & editing, Writing – original draft, Visualization, Validation, Supervision, Software, Resources, Project administration, Methodology, Investigation, Funding acquisition, Formal analysis, Data curation, Conceptualization. **Aijia Zhang:** Writing – review & editing, Investigation. **Fangtian Ying:** Funding acquisition. **Jue Wang:** Writing – review & editing, Supervision, Data curation. **Guanghui Huang:** Writing – review & editing, Supervision, Investigation, Funding acquisition.

## Declaration of competing interest

The authors declare that they have no known competing financial interests or personal relationships that could have appeared to influence the work reported in this paper.
